# Pontine Hemorrhage Mimicking Bell’s Palsy: Isolated Facial Nerve Palsy—A Case Report

**DOI:** 10.1155/crnm/2754979

**Published:** 2026-06-22

**Authors:** Rei Takeichi, Nobukazu Miyamoto, Haruka Takeshige-Amano, Wataru Sako, Nobutaka Hattori

**Affiliations:** ^1^ Department of Neurology, Juntendo University School of Medicine, 3-1-3 Hongo Bunkyo-ku, Tokyo, 113-0033, Japan, juntendo.ac.jp

**Keywords:** Bell’s palsy, MRI, pontine hemorrhage, slowly progressive, vascular risk factors

## Abstract

Bell’s palsy, the most common cause of idiopathic peripheral facial palsy, is typically attributed to viral reactivation; however, rare cases due to cerebrovascular disease must always be considered in the differential diagnosis. We report the case of a 75‐year‐old man with poorly controlled hypertension, diabetes mellitus, and a history of smoking who presented with progressive left‐sided facial paralysis over four days. Initially suspected to have Bell’s palsy due to the lower motor neuron pattern and gradual onset, further neuroimaging revealed a different etiology. T2^∗^‐weighted MRI showed a low‐signal area in the left pons, and a noncontrast CT confirmed a small hemorrhage. This atypical presentation, characterized by a gradual onset and absence of additional neurological symptoms, was attributed to focal compression of the facial nerve by the hematoma. The patient was treated conservatively with hemostatic agents, bed rest, and rehabilitation, leading to symptomatic improvement. His symptoms began to improve by the fourth day of hospitalization, and follow‐up imaging showed no hematoma expansion, with signs of absorption by Day 7. He was discharged after 15 days. This case highlights the diagnostic challenge in distinguishing Bell’s palsy from facial peripheral paralysis secondary to brainstem cerebrovascular events, particularly in patients with vascular risk factors. It underscores the importance of a “selective imaging strategy,” emphasizing that neuroimaging should be strongly considered in atypical or progressive cases, even when classic signs of a stroke are absent.

## 1. Introduction

Facial nerve palsy can arise from a wide range of etiologies affecting the facial nerve along its course. Bell’s palsy, an acute idiopathic peripheral facial paralysis, remains the most common cause [1]. Infectious diseases, particularly those associated with herpes simplex virus and varicella–zoster virus, are also well‐recognized causes [2]. Other etiologies include tumors, trauma, and inflammatory or autoimmune disorders affecting the facial nerve [3, 4]. In most cases, the lesion is located in the peripheral portion of the facial nerve after it exits the brainstem.

However, lesions within the brainstem may occasionally produce a clinical pattern that closely mimics idiopathic peripheral facial nerve palsy. Because the facial nerve nucleus and its fascicles are located within the pons, intra‐axial lesions in this region can present with peripheral facial palsy–like symptoms before the nerve exits the brainstem. In such cases, differentiation from idiopathic peripheral facial palsy can be challenging, particularly in the absence of additional neurological findings. Among brainstem lesions, ischemic stroke has occasionally been reported to present as isolated facial nerve palsy [5]. In contrast, pontine hemorrhage presenting solely with peripheral facial palsy–like symptoms appears to be extremely rare. This distinction is clinically important, as patients with isolated facial palsy are often initially evaluated in non‐neurological settings, where neuroimaging may not be immediately performed.

Here, we report a rare case of pontine hemorrhage in a patient with vascular risk factors, presenting with isolated peripheral facial palsy that progressively worsened over several days, closely mimicking Bell’s palsy and posing a diagnostic challenge.

## 2. Case Presentation

**FIGURE 1:**
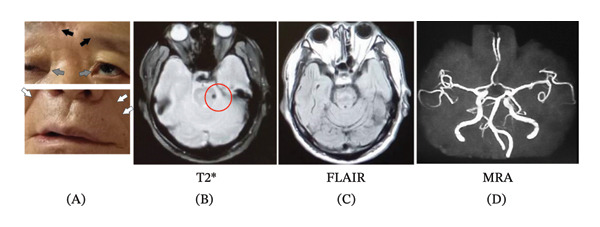
Facial view (A) and initial MRI image. (A) Facial view showing facial asymmetry. The left forehead lacks wrinkling (black arrow), the palpebral fissure is wider on the left than on the right (gray arrow), and the left nasolabial fold is flattened (white arrow). On MRI, a dot‐like hypointensity is observed in the left pons on T2^∗^‐weighted imaging ((B) red circle), and the same region shows a focal hypointense spot on FLAIR imaging (C). No vascular malformations are detected on MRA (D).

**FIGURE 2:**
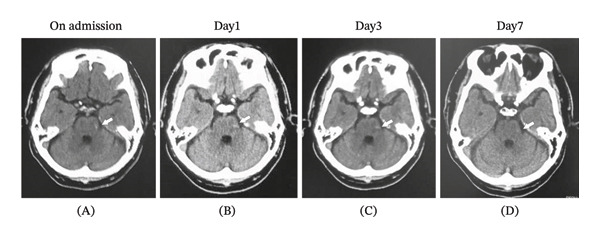
Temporal changes of the hematoma in the corresponding region on CT. The hematoma is indicated by arrows. From admission (A) to Day 1 (B), the hematoma becomes slightly more distinct; by Day 3 (C), its margin becomes less well defined, and by Day 7 (D), it becomes further indistinct.

**FIGURE 3:**
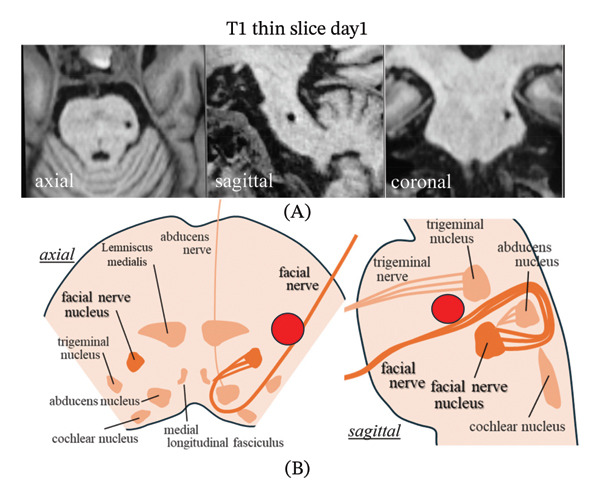
(A) Three planes (axial, sagittal, and coronal) of thin‐slice T1‐weighted images obtained on Day 1. (B) Schema of pontine level. The site of intracerebral hemorrhage is indicated by a red circle. Based on neuroimaging and clinical findings, the hematoma appears to compress the facial nerve emerging from the dorsal pons, while sparing the trigeminal nerve, vestibulocochlear nerve, cerebellar pathways, and the medial longitudinal fasciculus.

A 75‐year‐old man with poorly controlled hypertension (systolic blood pressure: 140–160 mmHg) and diabetes mellitus (HbA1c: 8.1%) had undergone shunt placement for normal pressure hydrocephalus. He developed progressive left‐sided facial paralysis over the course of 4 days. He initially noticed difficulty moving his mouth, which progressively worsened to an inability to wrinkle his forehead, resulting in complete left‐sided facial paralysis with no report of dry eyes, dry mouth, hearing impairment, facial sensory disturbance, or ocular motility impairment (Figure 1A). No skin lesions were observed around the external auditory canal, and sensation in the canal was intact. Bell’s palsy—a common diagnosis for peripheral facial paralysis in the absence of other neurological deficits—was initially suspected. However, further imaging studies revealed an alternative etiology. A T2^∗^‐weighted MRI demonstrated a low‐signal area in the left pontine region (Figure 1B–D), and a noncontrast head CT confirmed a small hemorrhage in the same area (Figure 2). This atypical presentation—characterized by slowly progressive peripheral facial paralysis without the sudden onset or additional cranial nerve involvement typically associated with cerebrovascular events—was attributed to gradual compression of the intrapontine facial nerve fascicles by the hematoma (Figure 3). Other laboratory findings were within normal limits; varicella–zoster virus serology showed positive IgG and negative IgM, indicating a past infection. Blood coagulation tests showed no abnormalities, and renal function was normal. Neuro‐otological examination demonstrated normal tympanometry and intact ossicular reflexes. Standard audiometric testing revealed high‐frequency hearing loss, considered consistent with age‐related changes. Blink reflex testing demonstrated absence of the left‐sided blink reflex, suggestive of facial nerve palsy. The patient received treatment with hemostatic agents (carbazochrome sodium sulfonate and tranexamic acid), bed rest, and rehabilitative therapy. His symptoms began to improve by the fourth day of hospitalization, and he was discharged after 15 days. Follow‐up CT scans revealed no further expansion of the hematoma, and by the seventh day after admission, signs of hematoma absorption were observed (Figure 2).

## 3. Discussion

Peripheral facial nerve palsy is most commonly caused by idiopathic Bell’s palsy or viral infections such as varicella–zoster virus. Consequently, patients presenting with isolated facial paralysis are frequently diagnosed and treated for peripheral facial nerve palsy in the initial clinical setting. However, lesions within the brainstem may occasionally produce a clinical pattern that closely mimics peripheral facial nerve palsy. This case underscores the diagnostic challenge in differentiating Bell’s palsy from brainstem lesions presenting with peripheral‐type facial palsy, particularly in patients with vascular risk factors such as hypertension and diabetes mellitus. In the present case, a small pontine hemorrhage presented as isolated facial paralysis without other neurological deficits, making the diagnosis particularly challenging. Although brainstem lesions are known to cause facial palsy, most previously reported cases are accompanied by additional neurological findings such as hearing disturbance, gaze palsy, sensory deficits, or long‐tract signs due to the compact arrangement of neural structures within the brainstem. In contrast, cases presenting with isolated peripheral‐type facial palsy symptoms are relatively uncommon.

Notably, even when the clinical presentation exhibits a classic lower motor neuron pattern involving the forehead, it can still originate from the brainstem. A recently reported case of ischemic stroke at the ventrolateral pontomedullary junction perfectly imitated isolated facial nerve palsy, highlighting that the absence of “central” features, such as forehead sparing, does not definitively exclude a brainstem event [6]. In cases of cerebral infarction, peripheral facial paralysis has been reported in association with anterior inferior cerebellar artery (AICA) syndrome [7], in which infarction within the AICA territory results in facial paralysis accompanied by hearing loss due to involvement of both the facial nerve nucleus and the cochlear nucleus. In addition, pontine infarctions involving the dorsal paramedian region may present as “eight‐and‐a‐half syndrome,” characterized by one‐and‐a‐half syndrome with ipsilateral facial nerve palsy [8]. However, reports of pontine infarction presenting solely with isolated peripheral‐type facial palsy are extremely limited.

In contrast, reports of hemorrhagic stroke presenting with isolated peripheral‐type facial palsy are even rarer. Min and Jung described 10 cases of peripheral facial palsy associated with cerebrovascular disease, including two cases of pontine hemorrhage [8]. However, both cases were accompanied by additional neurological findings, such as ataxia, hearing loss, nystagmus, or ocular movement disorders, suggesting the involvement of adjacent cranial nerve nuclei and cerebellar pathways [9]. Karadan [10] reported a case of isolated peripheral facial palsy due to pontine hemorrhage; however, the imaging was limited, making detailed anatomical evaluation difficult. In contrast, our case allowed for a more precise anatomical assessment due to the availability of comprehensive neuroimaging.

Another notable feature of this case was the gradual progression of facial palsy over several days, which further complicated the clinical diagnosis. This time course is typically observed in idiopathic or viral facial nerve palsy and may lead clinicians to initially exclude central causes. One possible explanation is that the small pontine hemorrhage gradually expanded. While significant perilesional edema may be unlikely in such a small hemorrhage, even minimal structural changes in the brainstem—where neural structures are densely packed—may potentially affect cranial nerve fascicles. Although the precise mechanism cannot be definitively confirmed, this anatomical vulnerability may partly explain the progressive course observed in this case.

From a clinical perspective, this case highlights an important diagnostic consideration. Patients with isolated facial paralysis are often initially evaluated by general practitioners or otolaryngologists, and neuroimaging may not always be performed at the initial visit. However, the necessity of brain imaging should be carefully evaluated based on patient risk factors. A recent large‐scale study in an emergency department setting indicated that while the overall yield of routine MRI for isolated facial palsy is low (around 3%), the presence of vascular risk factors or a history of malignancy significantly increases the likelihood of identifying a central lesion. This supports a “selective imaging strategy” where clinicians maintain a high index of suspicion for patients with such backgrounds [11]. In particular, when facial palsy shows progressive worsening, atypical features, or is associated with vascular risk factors, brain MRI should be considered to exclude central nervous system pathology, as it is more sensitive than CT for detecting small infarcts or hemorrhages [8].

From a practical clinical perspective, several “red flags” may help differentiate central causes from idiopathic peripheral facial nerve palsy. First, the absence of typical accompanying features of Bell’s palsy—such as dysgeusia, hyperacusis, lacrimation abnormalities, or sensory symptoms—may suggest a lesion within the brainstem or affecting the intra‐pontine fascicle. However, it should be noted that such findings are not uncommon in Bell’s palsy, as approximately two‐thirds of the patients may not exhibit sensory disturbances; therefore, this feature alone should be interpreted with caution. Second, the presence of vascular risk factors, including hypertension, diabetes mellitus, and a history of smoking, should raise suspicion for a cerebrovascular etiology. Third, the clinical course should be carefully evaluated. Gradual progression over several days is typical of Bell’s palsy and was also observed in the present case, which contributed to the diagnostic difficulty. In contrast, sudden onset with immediate maximal deficit is clearly atypical and may be more readily recognized as non‐Bell’s palsy. However, clinicians should remain cautious when the progression pattern is inconsistent, unusually prolonged, or accompanied by other subtle atypical features, as these findings may suggest a central etiology. Furthermore, when available, neuroimaging findings such as evidence of prior microbleeds or markers of small‐vessel disease—particularly from previous imaging studies—may provide additional support for a vascular etiology.

In conclusion, we report a rare case of pontine hemorrhage presenting as isolated peripheral‐type facial palsy that closely mimicked Bell’s palsy. This case expands the clinical spectrum of brainstem lesions presenting with facial palsy and emphasizes the importance of considering central causes in atypical presentations. This case may, therefore, serve as an educational example highlighting a potential diagnostic pitfall in the evaluation of isolated facial paralysis.

## Author Contributions

Conceptualization, data curation, investigation, and methodology: Rei Takeichi and Nobukazu Miyamoto; project administration, resources, software, and visualization: Rei Takeichi, Nobukazu Miyamoto, Haruka Takeshige‐Amano, and Wataru Sako; supervision and validation: Nobutaka Hattori; writing–original draft: Rei Takeichi and Nobukazu Miyamoto; writing–review and editing: all authors.

## Funding

The authors have not declared a specific grant for this research from any funding agency in the public, commercial, or not‐for‐profit sectors.

## Disclosure

This study was not commissioned and was internally peer reviewed.

## Ethics Statement

This study involves human participants and was approved by the Human Ethics Review Committee of Juntendo University School of Medicine (H15‐0213). Written informed consent was obtained from the patient for publication of this case report and accompanying images.

## Conflicts of Interest

The authors declare no conflicts of interest.

## Data Availability

Data used to support the findings of this study are available from the corresponding author upon reasonable request.
